# Does health worker performance affect clients’ health behaviors? A multilevel analysis from Bangladesh

**DOI:** 10.1186/s12913-019-4205-z

**Published:** 2019-07-24

**Authors:** Adrienne Epstein, Corrina Moucheraud, Haribondhu Sarma, Mahfuzur Rahman, Md. Tariqujjaman, Tahmeed Ahmed, Jeffrey Glenn, Thomas Bossert, Margaret E. Kruk

**Affiliations:** 1 Department of Epidemiology and Biostatistics, University of California, San Francisco, 550 16th Street, San Francisco, CA 94158 USA; 2 Department of Health Policy and Management, University of California Los Angeles, Fielding School of Public Health, Los Angeles, CA USA; 3 Nutrition and Clinical Services Division, icddr,b, Dhaka, Bangladesh; 4 Research School of Population Health, Australian National University, Acton, ACT 2601 Australia; 5 Department of Public Health, Brigham Young University, Provo, UT USA; 6 Department of Global Health and Population, Harvard T.H. Chan School of Public Health, Boston, MA USA

**Keywords:** health behavior, counseling, evidence-based practice, nutrition, Bangladesh

## Abstract

**Background:**

Suboptimal healthcare quality may be a barrier to achieving child health improvements, yet little is known about the relationship between provider compliance with evidence-based practices and client behavior change. We assess provider compliance in the context of infant and young child feeding (IYCF) counseling, its relationship with client IYCF behaviors in Bangladesh, and explore its potential determinants.

**Methods:**

We use data from a 2017 evaluation of an IYCF program that includes a health worker survey (*n* = 74), caregiver survey (*n* = 232), and direct service observation checklists of counseling sessions (*n* = 232 observations of 74 health workers). We assess the relationship between provider compliance with recommended IYCF counseling topics and behaviors (standardized to a 100-point scale) and three reported IYCF behaviors among clients using multi-level models with random effects at the health worker and sub-district (sampling) levels. We also evaluate whether health worker self-efficacy, satisfaction, and technical knowledge are associated with provider compliance.

**Results:**

Health worker compliance was significantly associated with reported exclusive breastfeeding for children under 6 months of age (adjusted odds ratio per 1 percentage point increase in counseling compliance score = 1.06, 95% CI 1.01, 1.12) and marginally associated with minimum dietary diversity (adjusted odds ratio per 1 percentage point increase in counseling compliance score = 1.05, 95% CI 1.00, 1.11). Counseling compliance was significantly and positively associated with both health worker self-efficacy and technical knowledge.

**Conclusions:**

We find evidence for an association between health worker compliance and client health behaviors; however, small effect sizes suggest that behavior change is multifactorial and affected by factors beyond care quality. Improvements to technical quality of care may contribute to desired health outcomes; but policies and programs seeking to change health behaviors through counseling may also wish to target upstream factors such as self-efficacy, alongside technical skill-building and knowledge, for maximum impact.

**Electronic supplementary material:**

The online version of this article (10.1186/s12913-019-4205-z) contains supplementary material, which is available to authorized users.

## Background

Across the globe, suboptimal healthcare quality poses a threat to improving population health outcomes. Annually, 5 million people in low- and middle-income countries die due to substandard healthcare quality [1]. Poor and inconsistent quality of care may jeopardize the realization of the Sustainable Development Goals [2]. In countries like Bangladesh, health care quality remains a barrier to realizing future gains. Bangladesh has made great strides in improving child health; for example, under 5 mortality has dropped from a rate of 144 deaths per 1000 live births in 1990 to 32 deaths per 1000 live births in 2017 [3]. However, a recent study estimated that over 90,000 deaths per year in Bangladesh are attributable to poor health care quality [1]. Across a number of low-income countries health care for children has been characterized by low and/or inconsistent quality [4–11]. Therefore, there is a critical need to identify specific gaps in quality and ways to address them.

Poor child nutrition causes a substantial burden of disease in Bangladesh and has been slow to improve: in 2014, the prevalence of stunting among children under age 5 was 36% and wasting was 14%, down from 41 and 16%, respectively, in 2011 [12, 13]. The burden of undernutrition in Bangladesh is largely attributed to inadequate infant and young child feeding (IYCF) behaviors [13]. For example, in 2014, it was estimated that only 55% of Bangladeshi children under 6 months of age were exclusively breastfed [14] per the World Health Organization recommendation for healthy growth and development [15]. Overall, this burden of child undernutrition poses a threat to the population’s future health and productivity [16].

One approach to improving child nutrition in Bangladesh is to provide quality counseling on IYCF practices for pregnant women and nursing mothers. An important dimension of high quality counseling is the counselor’s ability to follow evidenced-based practices, consisting of recommended IYCF topics and behaviors [2]. While the association between the receipt of high-quality counseling and improved client knowledge has been documented in various health care settings [9], there is scarce evidence showing whether this association extends to changing client behavior. Previous work has suggested that providers who are empathetic and emphasize client agency succeed in encouraging behavior change [17]. Additionally, models of health behavior change, such as the Social Cognitive Theory and the Information-Motivation-Behavioral Skills model, suggest that client knowledge plays a role in behavior change alongside other factors such as client self-efficacy, expectations, goals, and motivation [18–20]. Alive and Thrive, an IYCF initiative implemented at a large scale in Bangladesh, has demonstrated a positive impact on IYCF practices following the introduction of strengthened IYCF counseling – alongside mass media campaigns and advocacy activities [21–23] – but there is limited evidence on the relationship between actual quality of counseling and subsequent behavior change (IYCF practices).

While provider knowledge is critical for high-quality care, past research has identified a difference between provider knowledge and practice (“know-do gap”) [24–28]. To identify potential solutions to poor quality, it is therefore important to understand determinants of actual provider practice, including the role of factors beyond knowledge, such as self-efficacy, motivation and job satisfaction [29–32]. Health worker performance may also be associated with improvements in infrastructure, financial incentives, trainings, and management [27, 33].

**Fig. 1:**
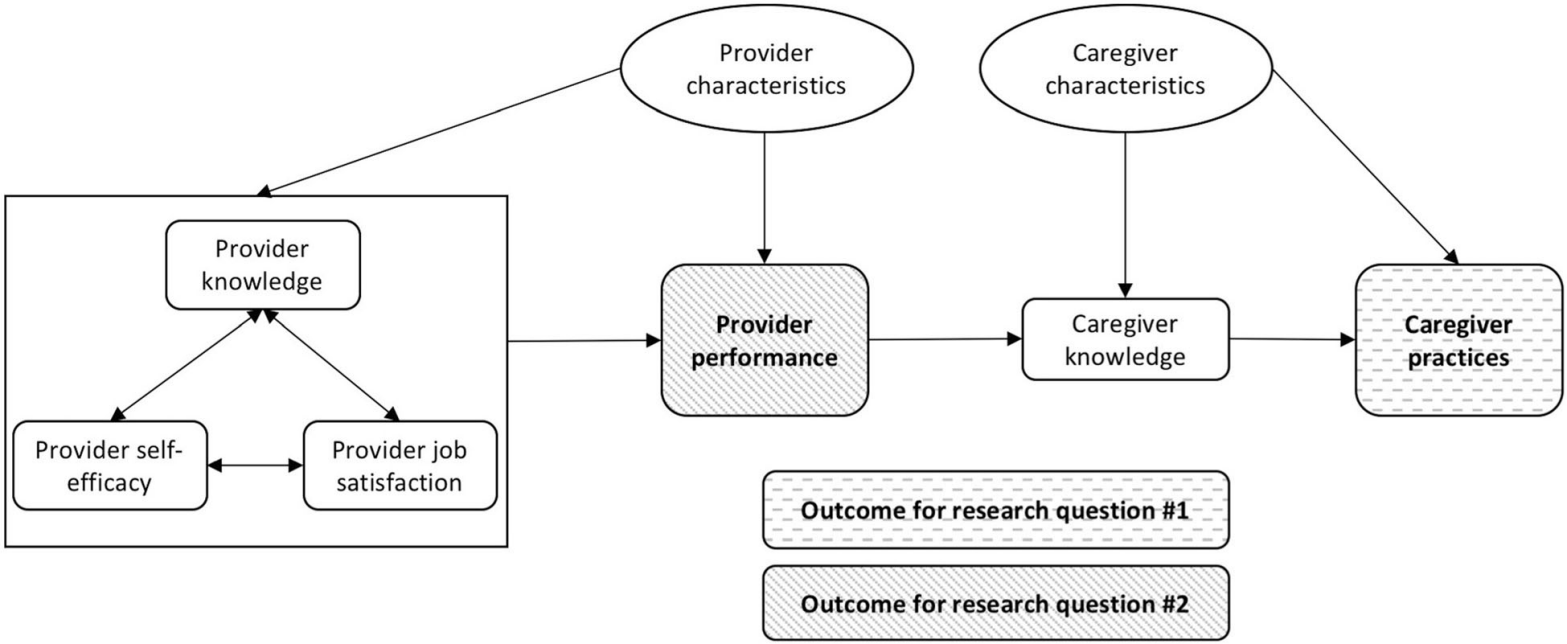
Framework linking provider performance and caregiver practices

In this paper, we focus on one dimension of health care quality: provider compliance with evidence-based practices. We first evaluate the relationship between health worker compliance with evidence-based practices (counseling caregivers on recommended IYCF topics and behaviors) and client breastfeeding and complementary feeding behavior among women in Bangladesh to determine whether compliant counseling leads to improvements in IYCF behaviors (see framework, Fig. 1). Additionally, we analyze whether health worker self-efficacy, satisfaction, and knowledge predict compliance in order to identify potential drivers of a key dimension of high quality care.

## Methods

### Study Sample

This study is a secondary analysis using data from the Alive and Thrive Sustainability Evaluation in Bangladesh, which aimed to examine determinants and dimensions of program sustainability after the conclusion of donor funding. In brief, 600 community health workers were randomly selected from 20 sub-districts in 10 of the 64 districts of Bangladesh, purposively selected from among those that participated in previous Alive and Thrive evaluations. These were volunteer community health workers supervised by the non-governmental organization BRAC, and included both Shasthya Shebikas (SS, general community health volunteers) and Pushti Shebikas (PS, community health volunteers working exclusively in IYCF). Volunteers were required to be female, married with no children under 5 years, and preferably had some schooling [34]. Each of these volunteers was supervised by an employed BRAC community health worker manager. These volunteers participated in a four-week training by BRAC that included best practices for IYCF counseling for pregnant and nursing women on a range of breastfeeding and complementary feeding practices. Each SS/PS was assigned to visit an average of 200–250 families per month within a select geographic area. Health workers were eligible for the study sample if they were an active provider of IYCF counseling, i.e. had a planned IYCF counseling visit within two days of initial contact by the research team.

Caregivers were then selected by randomly sampling the client lists from a random subset of 80 participating health workers (6 of whom had incomplete data so were not included in this analysis); eligible caregivers were women who were currently pregnant or had a child under the age of 24 months. In most cases, the sampled health worker had been providing counseling to the caregiver consistently on a monthly basis since she became pregnant. A total of 242 caregivers were sampled; 10 were excluded from this analysis due to missing data on key variables.

### Instruments

Three instruments were used: a health worker survey, a survey for caregivers, and a direct service observation checklist. All instruments were developed for the Alive and Thrive Sustainability Evaluation. They were based on those developed and validated during previous evaluations of Alive and Thrive [35, 36]. The surveys are available publicly [37]; additional modules used in this analysis are in Additional files 1 and 2. The health worker survey included questions about IYCF knowledge, job satisfaction, self-efficacy, and health worker characteristics. Caregivers were asked about their IYCF knowledge and practices, in addition to perceptions of service quality and background characteristics. The direct service observation checklist aimed to measure health worker counseling compliance and captured whether a set of evidence-based IYCF counseling activities were performed during the health worker-caregiver interaction. The checklist was designed for evaluations of Alive and Thrive [35, 36] based on the evidence-based guidelines for the BRAC/Alive and Thrive IYCF counseling service package, to assess technical content coverage and interpersonal communication quality.

### Data Collection

Experienced enumerators trained for the purpose of this study collected the data from January to May 2017. All data were collected and encrypted using SurveyCTO mobile data collection software. The caregiver was first surveyed, followed by direct service observation, and then the health worker was surveyed. Data were uploaded to a server daily and quality checks were performed regularly. In addition, a subsample of 5% of caregivers and health workers was resurveyed by study supervisors; test-retest agreement was 97% for both the health worker and caregiver surveys.

### Measures

To evaluate the relationship between provider compliance and reported client behavior change, we considered three outcomes measured in the parent study: minimum dietary diversity for children aged 6–24 months using 24-h dietary recall (asked of mothers with children aged 6–24 months), exclusive breastfeeding (EBF) among children under 6 months of age using 24-h dietary recall (asked of mothers with children under 6 months), and whether the caregiver self-reported breastfeeding their youngest child within 1 h of giving birth (asked of all mothers including pregnant women with children). The primary predictor of interest, provider compliance, was measured using a health worker service observation score, calculated by summing the number of tasks performed by the health worker and standardizing this to a 100-point scale (see Table 1, further details in Additional file 3). We included only technical content items in the counseling compliance score. Therefore, the score reflects the percent of technical items relevant to the counseling recipient (caregivers of children aged 0–6 months, 7–23 months, and pregnant women). Most workers had multiple service observations in the dataset (average number of observations 3.3, range 1 to 6); for those that had more than 1 score, the mean was calculated across these visits. For this aim we also adjusted for the mediating pathway of caregiver technical knowledge, measured through 14 survey items.

**Table 1:** Components of composite measures of knowledge, self-efficacy, and satisfaction

Composite measure	Item categories
Health worker counseling compliance	Counseling caregivers of children aged 0–6 months (19 items)
	Counseling caregivers of children aged 7–23 months (17 items)
	Counseling pregnant women (21 items)
Client knowledge score	Breastfeeding after birth (2 items)
	Exclusive breastfeeding (4 items)
	Breastfeed until at least 24 months (1 item)
	Timing of complementary feeding (2 items)
	Meal frequency (3 items)
	Feeding during and after illness (2 items)
Health worker knowledge score	Breastfeeding after birth (2 items)
	Exclusive breastfeeding (5 items)
	Solutions to common breastfeeding problems (3 items)
	Breastfeed until at least 24 months (1 item)
	Meal frequency (3 items)
	Feeding during and after illness (2 items)
	Encouraging complementary feeding (1 item)
Health worker self-efficacy	Confident can advise mothers about IYCF practices and can demonstrate BF and CF (3 items)
Health worker job satisfaction	Satisfied with job (1 item)
	Workload is manageable (1 item)
	Would like to remain in position (1 item)
	Adequate mentoring, supplies, and training opportunities (3 items)
	Work has positive impact (1 item)
	Feels recognized for work (1 item)

For our secondary aim – identifying determinants of provider compliance – the health worker service observation score was our primary outcome. We considered three predictors separately: health worker technical knowledge (a score based on 17 knowledge items), self-efficacy, and job satisfaction (see further details in Additional file 4). These scores (knowledge, self-efficacy and satisfaction) were standardized to a 100-point scale.

### Analysis

To estimate the relationship between reported caregiver behavior outcomes and health worker counseling performance, we specified separate mixed effects logistic regression models (with random intercepts at the sub-district and the health worker level to account for clustering). These models included the following caregiver-level predictors: caregiver age, religion (Muslim or not Muslim), years of schooling, wealth quintile (based on a household asset index score developed using principal component analysis), self-reported health status (5-point Likert scale), and number of health visits made by the caregiver to a health facility for any reason in the last year. We then re-specified the models including caregiver knowledge as a covariate, in order to determine whether quality of care was associated with behavior through pathways other than knowledge. We performed sensitivity analyses including the following health worker covariates: age, years of experience in the health sector, and years of schooling.

Models that aimed to explore determinants of health worker counseling compliance included random intercepts for the sub-district level and covariates for health worker age, years of experience, and years of schooling. All analyses were performed in Stata v14.

## Results

### Sample Characteristics

Clients were, on average, 25.0 years old and had completed 7.2 years of schooling (Table 2). Most (87.6%) were Muslim. Clients attended an average of 2.4 health visits with any provider during the year prior to the survey. The mean IYCF knowledge score was 68.0% (standard deviation [SD] 16.7). Approximately three-quarters of clients reported having breastfed their youngest child within 1 h of birth (75.3%) and, among those with children under 6 months of age, nearly three-quarters reported EBF (73.1%). Just over half of clients with children aged 6–24 months reportedly met minimum dietary diversity for this child over the past 24 h (55.1%).

**Table 2:** Characteristics of clients and health workers included in the sample

*Clients (caregivers of children under 24 months and pregnant women)*	*N* = 232
Age, mean (SD)	25.0 (5.5)
Years of schooling, mean (SD)	7.2 (3.2)
Muslim, % (n)	87.6% (204)
Number of health visits made in last year, mean (SD)	2.4 (0.9)
Total IYCF knowledge score (0–100), mean (SD)	68.0 (16.7)
Minimum dietary diversity among child aged 6–24 months, % (n)	55.1% (43)
EBF among children under 6 months, % (n)	73.1% (57)
Early initiation of breastfeeding for youngest child (within 1 h of birth), % (n)	75.3% (116)
*Health Workers*	*N* = 74
Age, mean (SD)	41.9 (10.7)
Years of experience in health sector, mean (SD)	9.7 (7.2)
Years of schooling, mean (SD)	5.5 (2.9)
Total IYCF knowledge score (0–100), mean (SD)	72.5 (11.9)
Counseling compliance score (0–100), mean (SD)	32.2 (15.8)
Self-efficacy score (0–100), mean (SD)	82.5 (17.6)
Satisfaction score (0–100), mean (SD)	82.1 (10.7)

SD standard deviation; IYCF infant and young child feeding; EBF exclusive breastfeeding

Surveyed health workers were an average of 41.9 years old, with 5.5 years of schooling, and had 9.7 years of experience working in the health sector. The average total IYCF knowledge score was 72.5% (SD 11.9) and average counseling compliance score was 32.2% (SD 15.8). The average self-efficacy score (82.5%) and satisfaction score (82.1%) were high.

### Correlates of caregiver IYCF outcomes

Adjusted models suggest that reported caregiver IYCF behavior was associated with provider counseling compliance (Table 3). These relationships were unchanged when caregiver knowledge score was included as a covariate, suggesting that health worker compliance may impact caregiver behavior through pathways other than the mediating pathway of IYCF knowledge (for models with caregiver knowledge score as a covariate, see Additional file 5). Women who received higher quality care were significantly more likely to report exclusive breastfeeding (adjusted odds ratio [AOR] = 1.06, 95% CI: 1.01, 1.12), and more likely to report minimum dietary diversity (AOR = 1.05, 95% CI: 1.00, 1.10) but the latter was only significant at the 10% level. Additional sensitivity analyses adjusting for health worker-level characteristics revealed similar results (Additional file 6).

**Table 3:** Relationship between counseling compliance and reported client IYCF practices

	Minimum dietary diversity (yes/no), *N* = 78	Exclusive breastfeeding until child 6 months of age (yes/no), *N* = 77	Early initiation of breastfeeding (within 1 hour of birth) (yes/no), *N* = 153
*Covariate*	Adjusted odds ratio (95% CI)	Adjusted odds ratio (95% CI)	Adjusted odds ratio (95% CI)
Health worker counseling compliance score (0-100)	1.04^†^ (1.00, 1.09)	1.06* (1.01, 1.12)	1.01 (0.98, 1.04)
Client age	1.00 (0.90, 1.11)	1.00 (0.90, 1.11)	0.94 (0.87, 1.02)
Client Muslim religion	0.36 (0.039, 3.34)	2.17 (0.31, 14.89)	1.04 (0.27, 4.00)
Client years of schooling	1.16 (0.90, 1.50)	0.94 (0.76, 1.16)	0.97 (0.85, 1.02)
Client wealth quintile	1.29 (0.79, 2.11)	1.05 (0.59, 1.87)	0.92 (0.67, 1.27)
Client self-reported health status	0.73 (0.38, 1.40)	1.16 (0.56, 2.42)	1.01 (0.64, 1.59)
Number of health visits client made last year	0.98 (0.53, 1.83)	0.59 (0.23, 1.51)	1.39 (0.91, 2.12)

Models include random effects for the sub-district and health worker

*IYCF* Infant and young child feeding, *CI* Confidence interval

^†^< 0.1, * < 0.05

### Correlates of health worker IYCF performance

Results from adjusted mixed effect linear models suggest that health worker counseling compliance was significantly and positively associated with health worker knowledge: a 1 percentage point increase in health worker knowledge was associated with 0.46 percentage point increase in health worker service observation score (95% CI: 0.21, 0.71) (Table 4). In addition, a 1 percentage point increase in health worker self-efficacy was associated with a 0.27 percentage point increase in health worker service observation score (95% CI: 0.076, 0.46). We did not find evidence of a relationship between health worker satisfaction and service observation score.

**Table 4:** Relationship between health worker characteristics (knowledge, self-efficacy, and satisfaction) and counseling performance (*N* = 74)

*Covariate*	Health worker counseling compliance score change, adjusted coefficient (95% CI)
Model 1	
**Health worker knowledge score (0–100)**	0.46*** (0.21, 0.71)
Health worker age	0.029 (−0.31, 0.37)
Health years of experience in health sector	−0.18 (− 0.71, 0.36)
Health worker years of schooling	− 0.56 (−1.56, 0.45)
Model 2	
**Health worker self-efficacy score (0–100)**	0.27** (0.076, 0.46)
Health worker age	−0.045 (− 0.39, 0.30)
Health years of experience in health sector	−0.38 (− 0.97, 0.22)
Health worker years of schooling	− 0.57 (−1.60, 0.46)
Model 3	
**Health worker satisfaction score (0–100)**	0.14 (−0.16, 0.44)
Health worker age	−0.073 (− 0.42, 0.28)
Health years of experience in health sector	−0.11 (0.68, 0.47)
Health worker years of schooling	−0.59 (−1.64, 0.46)

CI confidence interval

Models include random effects for sub-district (*N* = 20)

^†^ < 0.1, * < 0.05, ** < 0.01, *** < 0.001

## Discussion

Counseling caregivers and pregnant women on nutrition concepts and practices is commonly used as an intervention to improve IYCF practices [38]. However, to our knowledge, no previous studies have examined the relationship between health worker compliance with evidence-based counseling guidelines and women’s subsequent feeding practices. Using a unique survey design linking healthcare providers and clients, we examined this relationship and found evidence for a positive association between health worker compliance and improvements in IYCF behaviors. These relationships, however, had small effect sizes, reflecting the complexity of IYCF behavior change, which relies on many factors beyond technical quality of care.

The associations between quality of health worker compliance and client behavior persisted even when client knowledge was included in the models, suggesting that health worker behavior may influence client health behaviors through pathways other than knowledge. This may be because high-quality counseling does not differentially improve knowledge, but rather encourages the motivation to change behaviors (or the likelihood of reporting these behaviors). Alternatively, since clients may have seen these health workers previously, clients of high-quality counselors may have already experienced improvements to their IYCF knowledge in which case we observed residual effects on behavior but not the knowledge increases themselves. Or perhaps counselors with high service observation scores support clients beyond the counseling content for example by encouraging client self-efficacy or providing specific behavior counseling and goal-setting.

Importantly, this analysis also revealed low counseling compliance among health workers, with less than one-third of tasks completed on average. This score was substantially lower than the health worker IYCF knowledge score, which may reveal a know-do gap among health workers. The substandard quality of provider practice documented in this study is consistent with previous literature [9, 11, 39]. Notably, self-efficacy was significantly associated with improved performance scores. Interventions to improve health worker self-efficacy and confidence may lead to superior practice. These interventions may include mentorship and supportive supervision [33, 40]. Refresher trainings held regularly after the initial training may also improve practice [41, 42]. The suboptimal performance scores also may reflect workload challenges: given limited time with each caregiver, health workers may have prioritized some counseling items over others.

This study has several limitations. First, due to its cross-sectional nature we are unable to assess causality. For example, we found an association between health worker counseling compliance and self-efficacy; we cannot assess the directionality of this relationship which is likely bi-directional and should be the focus of future work. In addition, there may have been over-reporting of IYCF behaviors due to a simultaneous broader mass media campaign on breastfeeding and complementary feeding implemented by Alive and Thrive (with radio spots, television advertisements, and signs and billboards). Second, low sample sizes, particularly within some strata of clients and among health workers, may have limited our ability to detect associations. Third, our conclusions require assumptions regarding the consistency of provider compliance; in other words, we assume that counselors who performed well on the date of observation performed well in the past. In addition, the tasks the health worker performed in the one observed visit may not represent the full range of recommendations they made to the client over time. Fourth, the observed counseling compliance scores are likely higher than in reality due to the Hawthorne effect [43] although we observed most (99.2%) providers more than once. Finally, the composite scores employed here measuring knowledge, self-efficacy, and satisfaction may be imperfect in capturing the underlying constructs desired. To mitigate this, we used validated survey modules from previous studies.

## Conclusions

We found that, although provider practice was suboptimal, it was positively associated with clients’ behaviors in the context of IYCF counseling. Interventions that target provider confidence and self-efficacy may be effective in bettering health worker practice, and subsequently improving client outcomes. There is an urgent need to implement interventions that impact quality of counseling, beyond provider knowledge, to improve the status of child undernutrition in Bangladesh.

## Additional files

Additional file 1: Health worker survey: self-efficacy and job satisfaction module. Module from health worker survey used to generate self-efficacy and job satisfaction scores not included in the publicly available health worker survey. (DOCX 19 kb)

Additional file 2: Direct service observation checklist – Bangladesh. Direct service observation checklist used during counseling sessions with community health workers. (DOCX 35 kb)

Additional file 3: Full list of items included in service observation score. List of items from service observation checklist used to generate service observation score for each community health worker. (DOCX 18 kb)

Additional file 4: Full list of individual items included in knowledge, self-efficacy, and satisfaction composite measures. List of items from surveys used to generate knowledge, self-efficacy, and satisfaction composite measures for both health workers and caregivers. (DOCX 14 kb)

Additional file 5: Relationship between counseling quality and client IYCF practices adjusting for client knowledge score. Regression results evaluating relationship between counseling compliance and caregiver IYCF behaviors, adjusting for caregiver knowledge. (DOCX 13 kb)

Additional file 6: Relationship between counseling quality and client IYCF practices adjusting for health worker covariates (sensitivity analysis). Regression results evaluating relationship between counseling compliance and caregiver IYCF behaviors, adjusting for health worker characteristics. (DOCX 14 kb)

## Data Availability

All Alive and Thrive data will be publicly available through Dataverse. For further information, please contact the corresponding author.

## Abbreviations

EBF: Exclusive breastfeeding
IYCF: Infant and young child feeding
PS: Pushti Shebikas
SS: Shasthya Shebikas
