# Supplementary material for: Does health worker performance affect clients’ health behaviors? A multilevel analysis from Bangladesh
Source: BMC Health Serv Res. 2019 Jul 24;19:516. doi: 10.1186/s12913-019-4205-z (PMC6657138; doi:10.1186/s12913-019-4205-z)
Supplement: Supplementary file 1 — Health worker survey: self-efficacy and job satisfaction module. Module from health worker survey used to generate self-efficacy and job satisfaction scores not included in the publicly available health worker survey. (DOCX 19 kb) [file 12913_2019_4205_MOESM1_ESM.docx]

| **G. Self-efficacy and Job Satisfaction** | | | |
| --- | --- | --- | --- |
| The following questions ask about your level of satisfaction with your job. Please say whether you strongly disagree, disagree, neither agree nor disagree, agree, or strongly agree with the following statements. | | | |
| G1 | I am confident that I can advise the mothers or caregivers correctly about infant and young child feeding practice | Strongly disagree  Disagree  Neither agree or disagree  Agree  Strongly agree  No response | 1  2  3  4  5  99 |
| G2 | I am confident that I can correctly demonstrate complementary feeding procedures for children aged 6-23.9 months  (Complementary feeding is the process starting when breastmilk alone is no longer sufficient to meet the nutritional requirements of an infant and when other foods and liquids along with breastmilk are needed.) | Strongly disagree  Disagree  Neither agree or disagree  Agree  Strongly agree  No response | 1  2  3  4  5  99 |
| G3 | In general, I am satisfied with this job. | Strongly disagree  Disagree  Neither agree or disagree  Agree  Strongly agree  No response | 1  2  3  4  5  99 |
| G4 | I feel that my workload is manageable (not too heavy). | Strongly disagree  Disagree  Neither agree or disagree  Agree  Strongly agree  No response | 1  2  3  4  5  99 |
| G5 | If it were up to me, I would continue to do this work for quite some time. | Strongly disagree  Disagree  Neither agree or disagree  Agree  Strongly agree  No response | 1  2  3  4  5  99 |
| G6 | I have the mentoring and support I need to be successful in this position. | Strongly disagree  Disagree  Neither agree or disagree  Agree  Strongly agree  No response | 1  2  3  4  5  99 |
| G7 | I have the supplies and medications I need to perform my duties. | Strongly disagree  Disagree  Neither agree or disagree  Agree  Strongly agree  No response | 1  2  3  4  5  99 |
| G8 | I have access to adequate training opportunities to improve my skills. | Strongly disagree  Disagree  Neither agree or disagree  Agree  Strongly agree  No response | 1  2  3  4  5  99 |
| G9 | I believe the work I do has a positive impact on my community. | Strongly disagree  Disagree  Neither agree or disagree  Agree  Strongly agree  No response | 1  2  3  4  5  99 |
| G10 | I feel I am adequately recognized and rewarded for the work I do. | Strongly disagree  Disagree  Neither agree or disagree  Agree  Strongly agree  No response | 1  2  3  4  5  99 |
