# Supplementary material for: Does health worker performance affect clients’ health behaviors? A multilevel analysis from Bangladesh
Source: BMC Health Serv Res. 2019 Jul 24;19:516. doi: 10.1186/s12913-019-4205-z (PMC6657138; doi:10.1186/s12913-019-4205-z)
Supplement: Supplementary file 2 — Direct service observation checklist – Bangladesh. Direct service observation checklist used during counseling sessions with community health workers. (DOCX 35 kb) [file 12913_2019_4205_MOESM2_ESM.docx]

**Additional file 2**

Direct service observation checklist -- Bangladesh

**MODULE A: GENERAL INFORMATION**

Division:

Sub-district:

Village:

Name of observer:

Type of client (Select all that apply):

Mother of less than 6 months old [module B1] /

For 6-8 months: [module B2, B2.2a]

For 9-11 months: [module B2, B2.2b]

For 12-23 months: [module B2, B2.2c]

Pregnant woman in third trimester [module B3]

**MODULE B: OBSERVATION OF COUNSELING SESSION**

1. Greetings
   1. Health provider greets caregiver
   2. Asks how caregiver is doing

B1. For mothers of children aged less than 6 months

B1.1. Health provider counsels mother about exclusive breastfeeding

- 1. Duration (6 months)
  2. Benefits for baby
  3. Benefits for mother

B1.2. Position and attachment of breastfeeding

1. Assesses the position and attachment
2. Counsels about proper position and attachment
3. Demonstrates about how to maintain proper position and attachment

B1.3. Health provider reminds mother how to correctly assess milk supply

1. 6+ urinations per day
2. Child is gaining weight
3. Sleeps & plays well

B1.4. Health provider counsels on how to maintain good milk supply

a. Breastfeed frequently

b. Breastfeed for long duration

c. Explains common reasons for milk insufficiency

B1.5. Health provider teaches mother about expressing breastmilk

1. Why to express
2. How to express and store

B1.6. Health provider discusses timing for introducing complementary food

1. Importance of continuation of breastfeeding along with complementary (solid/semi-solid) foods
2. Explains how food groups can fill the gaps
3. Gives advice on age-appropriate feeding/ quantity
4. Gives advice on age-appropriate feeding/ Frequency
5. Safe preparation and storage of complementary foods

**B2. For mothers of children aged 6-23 months**

B2.1. Health provider reminds mother about the importance of continuation of breastfeeding with complementary (solid/semi-solid) foods

- 1. Duration of continuing breastfeeding (until 24 months or beyond)
  2. Benefits for baby
  3. Benefits for mother

B2.2. Health provider discusses complementary foods/ For 6-8 months: **)**

1. Asks about quantity of semi-solid/ solid food
2. Asks about frequency of semi-solid/ solid food
3. Counsels on correct quantity: half a bowl, bowl size 250 ml
4. Counsels on correct frequency: half a bowl two times a day )
5. For 9-11 months:
6. Asks about quantity of semi-solid/ solid food
7. Asks about frequency of semi-solid/ solid foods
8. Counsels on correct quantity: half a bowl, bowl size 250 ml
9. Counsels on correct frequency: half a bowl, three times a day and nutritious snacks 1-2 times
10. For 12-23 months:
11. Asks about quantity of semi-solid/ solid food
12. Asks about frequency of semi-solid/ solid foods
13. Counsels on correct quantity: one full bowl, bowl size 250 ml
14. Counsels on correct frequency: one full bowl, three times day and nutritious snacks 1-2 times
15. Explains how food groups can fill the gaps
16. Animal food plus 3 other varieties each day
17. Dark green leafy vegetable, ripe mango, ripe papaya, pumpkin
18. Thick lentil
19. Fried foods, ghee/ butter/ oil
20. Counsels about micronutrient powder
21. Safe preparation and storage of complementary foods
22. Counsels about spending time with the child and teaching the child to feed himself/ herself: For 9- 23months old child

B2.3. Health provider advises on water and sanitation

1. Keep water near place of child feeding
2. Keep soap near place of child feeding
3. Reminds mother to wash hands with soap each time before food preparation and feeding

**B3. For pregnant women in third trimester**

B3.1. Health care provider counsels about the mother’s nutrition during pregnancy

a. Tells the pregnant woman to eat an extra handful of food with all three

b. Tells her to eat more of: fish, eggs, meat, liver, dark green leafy vegetables, lentils, yellow fruits and vegetables, milk products and fried foods daily

c. Tells her to take one iron/folic acid tablet daily after her evening meal through the pregnancy

B3.2. Health provider talks about steps after delivery

- 1. Importance of skin-to-skin contact
  2. Initiation of breastfeeding (within 1 hour after delivery
  3. Special properties of colostrum, reasons why important
  4. No pre-lacteal feeding
  5. Optimal breastfeeding pattern
  6. Encourage breastfeeding on demand

B3.3. Health provider explains how EBF works

1. Duration (6 months)
2. No other foods or drinks
3. Benefits of EBF for baby
4. Benefits of EBF for mothers
5. Risks and hazards of not breastfeeding

B3.4. Health provider discusses position and attachment and milk expression

1. Importance of good positioning and attachment
2. Counsels about proper position and attachment
3. Demonstrates about how to maintain proper position and attachment
4. How to manually express breastmilk when needed

B3.5. Health provider asks mother about her intention to breastfeed

1. Identifies barriers she might have
2. Addresses barriers
3. Builds client's confidence in her ability to exclusively breastfeed

2. Health provider addresses mother's concerns

1. Health worker gives mother opportunity to ask questions
2. Health worker addresses all of mother’s stated questions
3. Plans a follow-up visit for the mother

3. Health worker checks any book/ card

- 1. Checks baby book/ card
  2. Writes in mother card

**MODULE C: SPECIFIC PROBLEMS & PROBLEM-SOLVING**

Did mother express any of these concerns?

Do not have milk/ milk is not sufficient

Flat and inverted nipple

Sore or cracked nipple

Engorgement, blocked duct

Plugged ducts and mastitis

Breast refusal

Mother is sick

Underweight/lightweight baby

Mother is malnourished

Mother is pregnant again

Mother is stressed

Mother is away from the baby

Twins

Fussy eating / refuse to eat

Ill child

Preventing diarrhea

Doctor advised to stop breastfeeding

Doctor advised to start tinned milk

C1. Do not have milk/ milk is not sufficient

1. Health worker provides solution
2. Compile a good feeding history
3. Observe and assess a breastfeed to check positioning and attachment
4. Support mother with correct positioning right from the first breastfeed
5. Listen to the mother and family to identify if there is any psychological problem or wrong beliefs
6. Encourage a good diet for the mother
7. Provide appropriate solutions and support
8. Ask mother about the number of wet diapers a baby produces each day
9. Assess the baby’s weight

C2. Flat and inverted nipple

1. Health worker provides solution
2. Build confidence for the mother by explaining that a baby suckles from the breast – not from the nipple; that the mother should breastfeed more because when the baby breastfeeds, he will stretch her nipple out
3. If inverted nipple is identified early during pregnancy, instruct mothers how to stretch the nipple or wear nipple shells
4. Encourage the mother to position the baby at the breast early after delivery and with patience before the milk comes in and her breasts are full
5. Show the mother how to make the nipple stretch out by herself before a feed
6. Show the mother how to shape the breast by supporting her breast from underneath with her fingers, and press the top of the breast gently with her thumb
7. If the breasts are full of milk: express breast milk and feed it to the baby with a cup. Continue to breastfeed the baby until the breasts are released
8. In complicated cases, use the syringe method

C3. Sore or cracked nipple

1. Health worker provides solution
2. Advice mother do not stop BF
3. Improve attachment. Begin to breastfeed on the side that hurts less
4. Vary BF positions
5. Let the baby come and attach to the breast by him/herself
6. Apply drops of breast milk to nipples and allow them to air dry
7. Do not use soap or cream on nipples
8. Do not wait until the breast is full to breastfeed
9. Continue to breastfeed the baby and do not use bottles

C4. Engorgement – blocked duct

1. Health worker provides solution
2. Apply cold compresses to breasts to reduce swelling
3. Breastfeed more frequently on demand, cue both day and night and offer both breasts
4. Improve attachment
5. Gentle stroking of breasts helps to stimulate milk flow (in the community, mothers often use a comb and gently comb the breast towards the nipple)
6. Press around the areola to reduce edema and to help baby to attach
7. Express milk to relieve pressure until baby can suckle
8. Express unused breast milk after each breastfeed

C5. Plugged Ducts and Mastitis

1. Health worker provides solution
2. Continue BF (if breast milk is not near the abscess let the baby feed more frequently and as often as s/he will)
3. Apply a warm compress (if mastitis is not detected)
4. Hold the baby in different positions, so that the baby‘s tongue/chin is close to the site of the plugged duct/mastitis (the reddish area). The tongue/chin will massage the breast and release the milk from that part of the breast
5. Ensure good attachment
6. Apply gentle pressure to the breast with of the palm of your hand, rolling fingers towards nipple
7. Then express milk or let the baby feed every two to three hours day and nigh
8. Rest (mother)
9. Drink more liquids (mother)
10. If there is no improvement in 24 hours, refer the mother to a health facility
11. Avoid tight clothing
12. Avoid holding the breast as scissors hold as it will prevent milk flow

C6. Breast refusal

1. Health worker provides solution
2. Advise mothers keep her baby close (skin to skin contact at all time, sleep with her baby)
3. Offer her breast whenever her baby is willing to suckle
4. Help her baby to take the breast by express breast milk into his mouth or position him well
5. Feed her baby the expressed breast milk by cup

C7. The mother is sick

1. Health worker provides solution
2. Continue breastfeeding when mother has a headache, pain in her back, a flu, diarrhea or other common diseases (malaria, anemia, cholera, etc.)
3. The mother should relax and drink more water to help her body recover
4. Go for a health check

C8. Underweight baby

1. Health worker provides solution
2. Support the baby to help him attach well to the breast, and hold the baby
3. Breastfeed long and slowly, keep the baby near the breast
4. In the first few weeks, the baby may not be able to suckle directly from mother‘s breast so the mother is encouraged to express breast milk and feed her baby by cup and spoon
5. If the baby sleeps too much, do not hold him tightly in order to wake him up for BF
6. Assure the breast is empty
7. Assess the frequency of feeding

C9. The mother has malnutrition

1. Health worker provides solution
2. She should eat more to get better
3. Supplement vitamin A within six weeks after delivery and micronutrient daily if available
4. Breastfeed more frequently

C10. The mother becomes pregnant again

1. Health worker provides solution
2. Encourage mother to continue BF is good for him/her in order to maintain good health, growth and development
3. Encourage mother to eat more meals per day to have good health, support BF and nurture the fetus

C11. The mother is stressed

1. Health worker provides solution
2. Explain that the quality of breast milk is not affected and milk secretion is not reduced if the mother is stressed. However, breast milk can temporarily come in infrequently
3. Encourage mother to continue BF, milk flow will recover
4. Often keep the mother and baby together if the mother agrees
5. Comfort and encourage the mother, enable her to talk and provide spiritual and practical support
6. Rest and breastfeed her baby
7. Drink warm water to relax and support breast milk secretion

C12. Mother is away from the baby

1. Health worker provides solution
2. Express and store breast milk to feed her baby at time of a normal breastfeed
3. Instruct the caregiver to feed the baby with safely expressed breast milk
4. Sleep with her baby in order to frequently BF at night when she is at home
5. If the mother can take her baby to her work place, continue BF frequently

C13. Twins

1. Health worker provides solution
2. Explain that the mother can exclusively breastfeed both babies if they are frequently breastfed and well attached to the breast
3. Explain that the more the baby suckles, the more breast milk is secreted
4. If the babies are unable to latch on immediately, help the mother to express her breast milk and feed them by a cup. Try to stimulate breast milk production early to make sure the breasts can secret enough milk for both babies

C14. Fussy eating/ refuse to eat

1. Health worker provides solution
2. Take an eating history
3. Explore clinical feeding disorder (unpredictable food refusal, fear of feeding, fussy eating)
4. Explain what can we do when the child is unpredictable food refusal: avoid letting the child witness family conflicts; if child interest in playing, being active overrides hunger cues, space meals and snacks for a while, try to wait until child feels hungry and asks for food; if child is more interested in what is going on around them than eating, reduce interaction such as turning off TV, no strolling while feeding; if child does not like parent’s control, apply child-led feeding
5. Give some solutions if child is fear based food-refusal: desensitization by gradual exposure; reassurance; creation of a pleasing eating environment; meal time is time for the child to learn and be loved
6. Explain that let the child eat diverse foods when starting CF; let the child expose to foods gradually for trust; repeat feeding the child for several times (10-15 times) for the child to like the food
7. Limit time at meals to around 20 minutes

C15_a. Feeding child during illness

1. Health worker provides solution
2. Health provider explains the importance of continued feeding during illness to the mother of child under 6 months of age
   - 1. Remind mother the importance of exclusive breastfeeding
     2. Instruct mother to breastfeed more frequently
3. Health provider explains the importance of continued feeding during illness to the mother of child aged 6 months or more
4. Instruct mother to breastfeed more frequently
5. Instruct the mother to feed the child more frequently
6. Explain to the mother how much water should be fed to the child
7. Describe to the mother types of food that should be eaten during illness (nutrient-rich and energy-rich) and what types of food that should be avoided (high-fiber and nutrient poor)

C15_b. Preventing diarrhea

1. Health worker provides solution
2. Exclusive breastfeeding in first six months and continuation of breastfeeding to two years
3. Complementary feeding after six months
4. Hygienic practices for both food and utensils
5. Hand washing techniques
6. Proper way to dispose of waste
7. Vaccinations

C16 (a and b). Doctor advise to stop exclusive breastfeeding (child <6 months)

1. Health worker provides solution

a. Health worker explain the dangers of not breastfeeding exclusively

b. Encourage mother to breastfeed frequently and stop all other foods and liquids

**MODULE D: OBSERVATION OF INFRASTRUCTURE AND MATERIAL USE**

**1. Alive and Thrive program materials**

- 1. Job aids are available for use
  2. Job aids are in good condition
  3. Job aids are used

**2. Time**

1. How much time health worker spends discussing infant and young child feeding
