# Supplementary material for: Does health worker performance affect clients’ health behaviors? A multilevel analysis from Bangladesh
Source: BMC Health Serv Res. 2019 Jul 24;19:516. doi: 10.1186/s12913-019-4205-z (PMC6657138; doi:10.1186/s12913-019-4205-z)
Supplement: Supplementary file 3 — Full list of items included in service observation score. List of items from service observation checklist used to generate service observation score for each community health worker. (DOCX 18 kb) [file 12913_2019_4205_MOESM3_ESM.docx]

**Additional file 3**

**Full list of items included in service observation score**

| **Client Type** | **Checklist items** |
| --- | --- |
| Caregivers of child aged 0-6 months | 1. Health provider reminds mother about the benefits of EBF 2. Duration (6 months) 3. Benefits for baby 4. Benefits for mother 5. Health provider assesses and counsels on position and attachment 6. Assesses 7. Counsels 8. Demonstrates about how to maintain proper position and attachment 9. Health provider reminds mother how to correctly assess milk supply 10. 6+ urinations per day 11. Growing well 12. Sleeps and plays well 13. Health provider counsels on how to maintain good milk supply 14. Breastfeed frequently 15. Breastfeed for long duration 16. Explains common reasons for milk insufficiency 17. Health provider teaches mother about expressing breastmilk 18. Why to express 19. How to express and store 20. Health provider discusses timing for introducing complementary food 21. Importance of sustaining breastfeeding while supplementing with solid/semi-solid foods 22. Explains how food groups can fill the gaps 23. Gives advice for age-appropriate feeding: quantity 24. Gives advice for age-appropriate feeding: frequency 25. Safe preparation and storage of complementary foods |
| Caregivers of child aged 7-23 months | 1. Health provider reminds mother about the importance of sustaining breastfeeding while supplementing with solid/semi-solid foods 2. Duration of continuing breastfeeding (until 24 months or beyond) 3. Benefits for baby 4. Benefits for mother 5. Health provider discusses complementary foods 6. Asks about quantity of semi-solid/solid food (appropriate for child's age) 7. Counsels on correct quantity of semi-solid/solid food (appropriate for child's age) 8. Asks about frequency of semi-solid/solid food (appropriate for child's age) 9. Counsels on correct frequency of semi-solid/solid food (appropriate for child's age) 10. Explains how food groups can fill the gaps 11. Animal food plus 3 other varieties each day 12. Counsels on micronutrient powder 13. Safe preparation and storage of complementary foods 14. Counsels about spending time with the child and teaching child to food himself   3. Health provider advises on water and sanitation   1. Keep water near place of child feeding 2. Keep soap near place of child feeding 3. Reminds mother to wash hands with soap each time before food preparation and feeding |
| Pregnant woman | 1. Health provider counsels about the mother’s nutrition during pregnancy  a. Tells the pregnant woman to eat an extra handful of food with all three meals  b. Tells her to eat more of: fish, eggs, meat, liver, dark green leafy vegetables, lentils, yellow fruits and vegetables, milk products and fried foods daily  c. Tells her to take one iron/folic acid tablet daily after her evening meal throughout the pregnancy  2. Health provider talks about steps after delivery  a. Importance of skin-to-skin contact  b. Initiation of breastfeeding (within 1 hour of delivery)  c. Special properties of colostrum, reasons why important  d. No pre-lacteal feeding  e. Optimal breastfeeding pattern  f. Encourage breastfeeding on demand  3. Health provider explains how EBF works  a. Duration (6 months)  b. No other food or drinks  c. Benefits of EBF for baby  d. Benefits of EBF for mothers  e. Risks and hazards of not breastfeeding  4. Health provider discusses position and attachment and milk expression  a. Importance of good positioning and attachment  b. Counsels about proper position and attachment  c. Demonstrates about how to maintain proper position and attachment  d. How to manually express breastmilk when needed  5. Health provider asks mother about her intention to breastfeed  a. Identifies barriers she might have  b. Addresses barriers  c. Builds client's confidence in her ability to exclusively breastfeed |
