# Supplementary material for: Does health worker performance affect clients’ health behaviors? A multilevel analysis from Bangladesh
Source: BMC Health Serv Res. 2019 Jul 24;19:516. doi: 10.1186/s12913-019-4205-z (PMC6657138; doi:10.1186/s12913-019-4205-z)
Supplement: Supplementary file 4 — Full list of individual items included in knowledge, self-efficacy, and satisfaction composite measures. List of items from surveys used to generate knowledge, self-efficacy, and satisfaction composite measures for both health workers and caregivers. (DOCX 14 kb) [file 12913_2019_4205_MOESM4_ESM.docx]

**Additional file 4**

**Full list of individual items included in knowledge, self-efficacy, and satisfaction composite measures**

| **Composite measure** | **Individual items** |
| --- | --- |
| Client knowledge: Breastfeeding | 1. Child should be breastfed immediately after birth  2. Child should be given colostrum  3. Child should be given expressed breastmilk when mother is away  4. List at least one reason child under 6 months should be exclusively breastfed  5. Child under 6 months should not be given water in hot weather  6. Child should be breastfed until 24 months of age  7. Mother should continue breastfeeding even if pregnant |
| Client knowledge: Complementary feeding | 1. Child should start receiving liquids at 6 months  2. Child should start receiving foods at 6 months  3. Meal frequency at 6-8.9 months (at least 2 meals)  4. Meal frequency at 9-11.9 months (at least 3 meals)  5. Meal frequency at 12-23.9 months (at least 3 meals)  6. Feeding during illness  7. Feeding after illness |
| Client knowledge: Combined | All 14 items above |
| Health worker knowledge: Breastfeeding | 1. Child should be breastfed immediately after birth  2. Child should be given colostrum  3. Child should be given expressed breastmilk when mother is away  4. List at least one reason child under 6 months should be exclusively breastfed  5. Child under 6 months should not be given water in hot weather  6. Child should be breastfed until 24 months of age  7. Mother should continue breastfeeding even if pregnant  8. Most common reason for sore/painful breasts is poor positioning and attachment  9. List at least 1 way a mother can tell child is getting enough milk  10. Mother should breastfeed more often/frequently if she thinks her child is not getting enough breastmilk  11. Child should be exclusively breastfed until 6 months |
| Health worker knowledge: Complementary feeding | 1. Meal frequency at 6-8.9 months (at least 2 meals)  2. Meal frequency at 9-11.9 months (at least 3 meals)  3. Meal frequency at 12-23.9 months (at least 3 meals)  4. Feeding during illness  5. Feeding after illness  6. List at least one way to encourage complementary feeding |
| Health worker knowledge: Combined | All 17 items above |
| Health worker self-efficacy | 1. Is confident they can advise mothers correctly about IYCF practices  2. Is confident they can demonstrate complementary feeding for mothers of children aged 6-23 months  3. Is confident they can demonstrate positioning for breastfeeding |
| Health worker job satisfaction | 1. Is satisfied with job  2. Feels workload is manageable  3. Would continue to remain in position for quite some time  4. Has mentoring and support needed to be successful  5. Has supplies and medications needed to perform duties  6. Has access to adequate training opportunities to improve skills  7. Believes work has a positive impact on the community  8. Feels adequately recognized and rewarded for work |
