# Supplementary material for: Does health worker performance affect clients’ health behaviors? A multilevel analysis from Bangladesh
Source: BMC Health Serv Res. 2019 Jul 24;19:516. doi: 10.1186/s12913-019-4205-z (PMC6657138; doi:10.1186/s12913-019-4205-z)
Supplement: Supplementary file 5 — Relationship between counseling quality and client IYCF practices adjusting for client knowledge score. Regression results evaluating relationship between counseling compliance and caregiver IYCF behaviors, adjusting for caregiver knowledge. (DOCX 13 kb) [file 12913_2019_4205_MOESM5_ESM.docx]

**Additional file 5**

**Relationship between counseling quality and client IYCF practices adjusting for client knowledge score**

|  | **Minimum dietary diversity (yes/no), n=78** | **Exclusive breastfeeding until child 6 months of age (yes/no), n=77** | **Breastfed within 1 hour of birth (yes/no), n=153** |
| --- | --- | --- | --- |
|  | **Adjusted odds ratio (95%) CI** | **Adjusted odds ratio (95%) CI** | **Adjusted odds ratio (95%) CI** |
| Health worker counseling compliance score (0-100) | 1.05^†^ (1.00, 1.10) | 1.06* (1.01, 1.12) | 1.01 (0.98, 1.04) |
| Client knowledge score (0-100) | 1.02 (0.98, 1.06) | 1.05* (1.00, 1.09) | 1.05** (1.02, 1.07) |
| Client age | 1.00 (0.98, 1.06) | 0.96 (0.85, 1.08) | 0.91* (0.84, 0.99) |
| Client Muslim religion | 0.32 (0.036, 2.82) | 1.16 (0.16, 8.20) | 0.48 (0.13, 1.82) |
| Client years of schooling | 1.17 (0.90, 1.52) | 0.92 (0.73, 1.15) | 0.92 (0.80, 1.06) |
| Client wealth quintile | 1.24 (0.76, 2.02) | 1.08 (0.61, 1.90) | 0.97 (0.70, 1.35) |
| Client self-reported health status | 0.69 (0.36, 1.35) | 0.97 (0.44, 2.17) | 0.81 (0.51, 1.29) |
| Number of health visits client made last year | 1.04 (0.56, 1.94) | 0.64 (0.25, 1.66) | 1.51^†^ (0.98, 1.29) |
| Models include random effects for the sub-district and health worker.  ^†^ < 0.1, * < 0.05, ** < 0.01, *** < 0.001 | | | |
